# Expanding Diversity in Molecular Structures and Functions of the IL-6/IL-12 Heterodimeric Cytokine Family

**DOI:** 10.3389/fimmu.2016.00479

**Published:** 2016-11-04

**Authors:** Hideaki Hasegawa, Izuru Mizoguchi, Yukino Chiba, Mio Ohashi, Mingli Xu, Takayuki Yoshimoto

**Affiliations:** ^1^Department of Immunoregulation, Institute of Medical Science, Tokyo Medical University, Tokyo, Japan

**Keywords:** IL-6, IL-12, IL-23, IL-27, IL-30, IL-35, IL-39, IL-Y

## Abstract

The interleukin (IL)-6/IL-12 family cytokines have pleiotropic functions and play critical roles in multiple immune responses. This cytokine family has very unique characteristics in that they comprise two distinct subunits forming a heterodimer and each cytokine and receptor subunit shares with each other. The members of this cytokine family are increasing; currently, there are more than six cytokines, including the tentatively named cytokines IL-Y (p28/p40), IL-12 (p35/p40), IL-23 (p19/p40), IL-27 [p28/Epstein–Barr virus-induced protein 3 (EBI3)], IL-35 (p35/EBI3), and IL-39 (p19/EBI3). This family of cytokines covers a very broad range of immune responses, including pro-inflammatory responses, such as helper T (Th)1, Th2, and Th17, to anti-inflammatory responses, such as regulatory T (Treg) cells and IL-10-producing Treg cells. IL-12 is the first member of this family, and IL-12, IL-23, and IL-27 are mainly produced by activated antigen-presenting cells, such as dendritic cells and macrophages. IL-12 plays a critical role in the promotion of Th1 immune responses by inducing interferon-γ production to combat pathogens and malignant tumors. IL-23 induces IL-17 production and is necessary to maintain pathogenic Th17 cells that cause inflammatory and autoimmune diseases. IL-27 was initially reported to play a critical role in promotion of Th1 differentiation; however, subsequent studies revealed that IL-27 has broader stimulatory and inhibitory roles by inducing IL-10-producing Treg cells. IL-35 is produced by forkhead box P3^+^ Treg cells and activated B cells and has immunosuppressive functions to maintain immune tolerance. The most recently identified cytokine, IL-39, is produced by activated B cells and has pro-inflammatory functions. The cytokine tentatively named IL-Y seems to have anti-inflammatory functions by inhibiting Th1 and Th17 differentiation. In addition, individual cytokine subunits were also shown to have self-standing activities. Thus, promiscuity within the IL-6/IL-12 family cytokines complicates structural and functional clarification and assignment of individual cytokines. A better understanding of the recent advances and expanding diversity in molecular structures and functions of the IL-6/IL-12 family cytokines could allow the creation of novel therapeutic strategies by using them as tools and targeted molecules.

## Introduction

**Figure 1:**
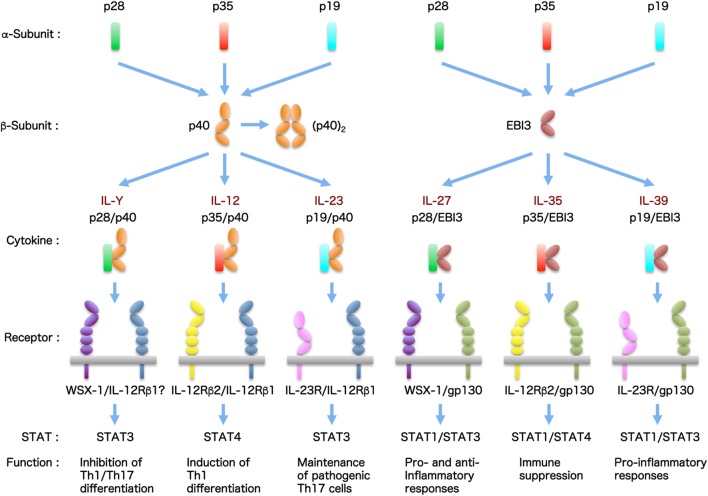
**The IL-6/IL-12 family cytokines**. The IL-6/IL-12 family cytokines have pleiotropic functions and play critical roles in multiple immune responses. This cytokine family has very unique characteristics, because they are composed of two distinct subunits forming a heterodimer and each cytokine and receptor subunit shares with each other. The members of this cytokine family are increasing. Currently, there are more than six cytokines, including tentatively named cytokines.

Generally, cytokines are grouped into distinct families depending on the differences in homology among amino acid sequences and structural characteristics. These characteristics include the protein higher-order structure and usage of certain membrane-bound cytokine β-receptors for signal transduction. In particular, the interleukin (IL)-6/IL-12 family cytokines have unique structure properties in that they comprise distinct α-subunits and β-subunits forming a heterodimer and they share cytokine subunits and cellular receptors with each other (1–3). The α-subunit is a four-helix bundle, long-chain structure similar to the type I cytokine, IL-6, and includes IL-23/IL-39p19, IL-27/IL-30p28, and IL-12/IL-35p35. The β-subunit is composed of two tandem fibronectin type III domains that form a cytokine-binding homology region and an N-terminal immunoglobulin (Ig) domain. This subunit is structurally related to the non-signaling receptor of IL-6, soluble IL-6 α-receptor (sIL-6Rα), and includes IL-12/IL-23p40 and IL-27/IL-35/IL-39 Epstein–Barr virus-induced protein 3 (EBI3). By promiscuous pairing between these α-subunits and β-subunits, more than six heterodimeric cytokines were reported to exist (Figure 1).

Interleukin-6 is one of the most important inflammatory cytokines; it is unique in signaling through not only membrane-bound IL-6Rα but also sIL-6Rα together with ubiquitously expressed β-receptor glycoprotein 130 (gp130) (4, 5). The former is called IL-6 classic signaling through membrane-bound IL-6Rα together with gp130 (4, 5). In contrast, the latter is called IL-6 *trans*-signaling by IL-6 bound with sIL-6Rα and IL-6/sIL-6Rα (also designated Hyper-IL-6), which is generated under pathological conditions, and signals only through gp130 as a receptor (4, 5). Because the IL-6/sIL-6Rα complex is similar in molecular structure to the IL-12 family heterodimeric cytokines and has homology with the IL-12 family cytokines, IL-6 and the IL-12 family cytokines are generally called IL-6/IL-12 family cytokines. This family of cytokines has emerged as key players in promotion and suppression of multiple immune responses under physiological and pathological situations.

Interleukin-12 is the first member of this family and consists of two subunits, p35 connected to an intra-chain disulfide with soluble α-receptor p40 (6, 7). p40 also binds to another β-subunit, p19, by the disulfide bridge to form the second heterodimeric cytokine called IL-23 (8), whereas p40 alone forms the antagonistic or agonistic disulfide-connected homodimer p80 (9). IL-12 and IL-23 engage a heterodimeric receptor complex of IL-12Rβ1 and IL-12Rβ2 and of IL-12Rβ1 and IL-23R, respectively, and share IL-12Rβ1 for signaling (10). IL-27 consists of two subunits, p28 (IL-30) and a soluble α-receptor EBI3, and signals *via* a receptor complex of WSX-1 and gp130 (11). IL-27 differs from the other IL-6/IL-12 family cytokines in that its subunits are not covalently linked. IL-35 shares the EBI3 of IL-27 and signals *via* four different receptor complexes: IL-12Rβ2/gp130, IL-12Rβ2/IL-12Rβ2, gp130/gp130, and IL-12Rβ2/WSX-1 (12, 13). IL-39, which was most recently discovered, consists of EBI3 and p19 and signals *via* IL-12Rβ1 and IL-23R (14). p40 also binds to p28 to form p28/p40, tentatively called IL-Y, but this complex was demonstrated to be an antagonist to the signaling by IL-12 and IL-27 (15, 16). In addition, biological activities of monomeric forms of p28 (17), EBI3 (18), and p19 (19) and generation of soluble receptors of WSX-1 (20) and IL-23R (21) as antagonists to IL-27 and IL-23, respectively, were reported as well.

**Figure 2:**
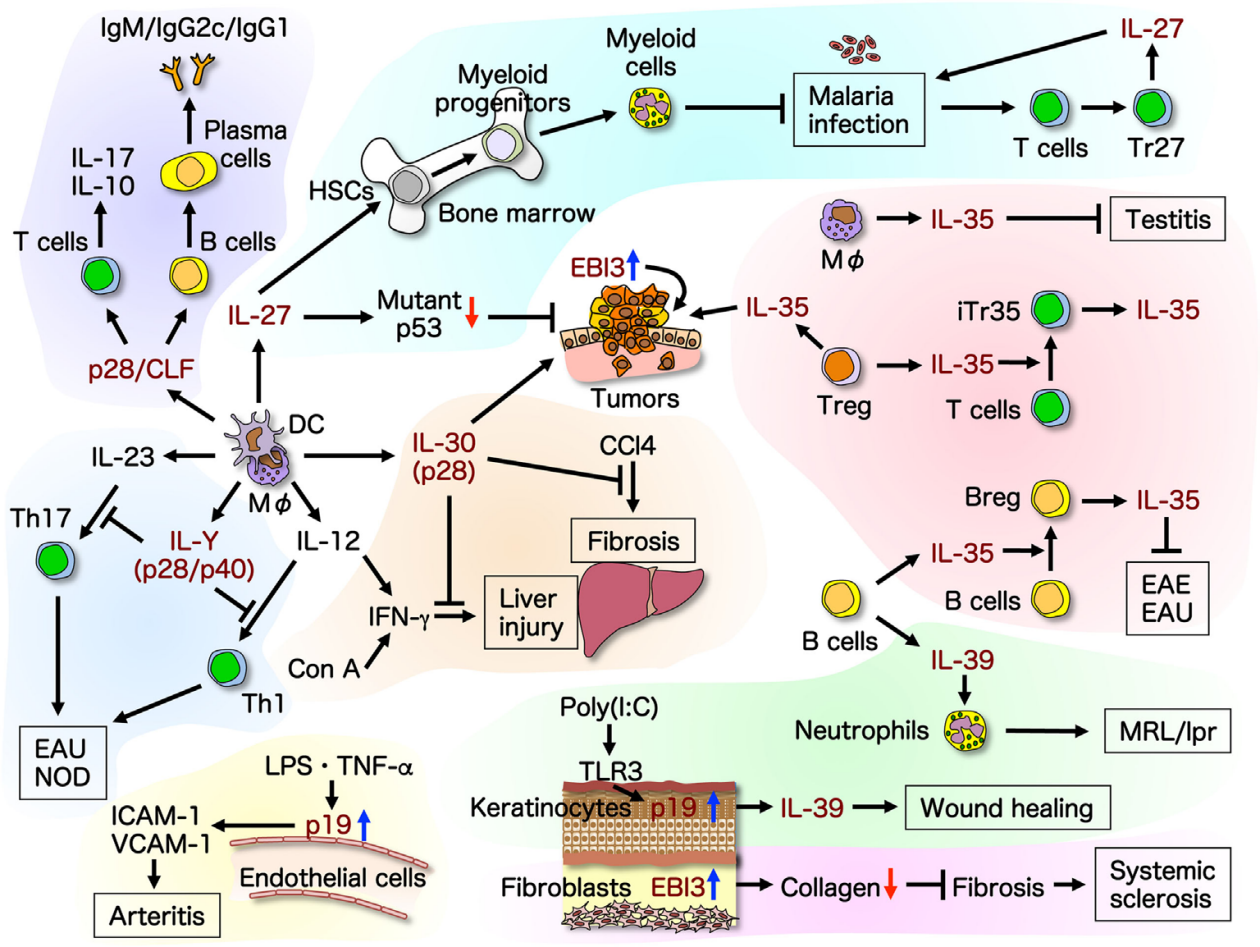
**Recent advances in molecular structures and functions of the IL-6/IL-12 family cytokines**. Promiscuity within the IL-6/IL-12 family cytokines complicates the structural and functional clarification and assignment of individual cytokines. The roles of this family of cytokines are expanding, and individual cytokine subunits have been shown to have self-standing activities. This illustrates the recent advances in molecular structures and functions of the IL-6/IL-12 family cytokines. Up-arrow in blue and down-arrow in red mean upregulation and downregulation of expression, respectively. Breg, regulatory B; CCl4, carbon tetrachloride; CLF, cytokine-like factor 1; Con A, concanavalin A; DC, dendritic cell; EAE, experimental autoimmune encephalomyelitis; EAU, experimental autoimmune uveitis; HSC, hematopoietic stem cell; ICAM-1, intracellular adhesion molecule-1; LPS, lipopolysaccharide; MΦ, macrophage; NOD, non-obese diabetic; TLR, toll-like receptor; TNF, tumor necrosis factor; VCAM-1, vascular cell adhesion molecule-1.

In this review, we summarize and discuss the recent advances and expanding diversity in molecular structures and functions of the IL-6/IL-12 family cytokines (Figure 2) to enable a better understanding of them and their use as tools and targeted molecules for novel therapeutic strategies.

## Interleukin-27

Interleukin-27 is a multifunctional cytokine with both pro-inflammatory and anti-inflammatory properties (11, 22). Although initial studies demonstrated the critical role of IL-27 in the induction of helper T (Th)1 differentiation (23–27), subsequent studies revealed that IL-27 has broader stimulatory and inhibitory roles in T-cell proliferation, differentiation, cytokine production, and effector functions (11, 22). IL-27 prevents the development of autoimmune diseases, such as experimental autoimmune encephalomyelitis and arthritis, by inhibiting Th17 differentiation and immune responses (28). In addition, IL-27 not only promotes protective Th1 immune responses against pathogens but also suppresses them, limiting excessive inflammation (29, 30) in part by IL-27-mediated induction of IL-10-producing regulatory T (Treg) cells (31, 32).

Host control of infections mainly induces Th1 responses in which interferon (IFN)-γ, IL-12, and tumor necrosis factor-α play critical roles in eliciting protective immunity against parasites, such as *Toxoplasma* and *Plasmodium*. However, continuing activation of effector immune cells provokes overproduction of pro-inflammatory cytokines, leading to exacerbated inflammatory reaction and lethality. Anti-inflammatory cytokines, such as IL-10 and IL-27, were demonstrated to be important for limiting the exacerbated protective Th1 responses (33–36). Moreover, malaria infection inhibits immune responses to the parasite itself, and CD4^+^ T cells from malaria-infected mice and humans have defects in the ability to produce IL-2 in response to T-cell receptor stimulation (37). Recently, a unique subpopulation of malaria-specific CD4^+^ T cells was revealed to produce IL-27 in response to T-cell receptor stimulation, subsequently inhibiting IL-2 production and clonal expansion of other T cells (38). The IL-27-producing CD4^+^ T cells are forkhead box P3^−^ CD11a^+^CD49d^+^ malaria antigen-specific CD4^+^ T cells, and they are distinct from IFN-γ-producing Th1 or IL-10-producing Treg cells (38). Thus, IL-27-producing regulatory CD4^+^ T cells, designated Tr27 cells, play a critical role in the regulation of protective immune responses against malaria parasites.

During infection, cytokines play pivotal roles in both induction of protective immunity and exacerbation of inflammatory responses. Multiple mechanisms to induce protection of the host from infection have been reported. Emergency myelopoiesis, which is one of these protective responses, is inflammation-induced hematopoiesis to replenish myeloid cells in the periphery, which is critical for controlling infection with pathogens (39, 40). Transgenic mice expressing IL-27 previously showed enhanced myelopoiesis in the bone marrow and extramedullary hematopoiesis in the spleen (41). Hematopoietic stem cells express both IL-27R subunits, such as WSX-1 and gp130 (41). IL-27 was recently revealed to directly act on hematopoietic stem cells and promote their expansion and differentiation to myeloid progenitor cells *in vitro* and *in vivo* in synergy with stem cell factor (42). In addition, it was demonstrated that IL-27 plays an important role in the control of infection with *Plasmodium berghei*-attenuated variant XAT. IL-27, which is produced through IFN-γ production during malaria infection, promotes expansion and differentiation of hematopoietic stem cells to myeloid progenitors and mobilizes them into the spleen, resulting in enhanced myelopoiesis with increased numbers of mature myeloid cells such as neutrophils (42). Thus, IL-27 is one of the limited unique cytokines directly acting on hematopoietic stem cells and promoting their expansion and differentiation into myeloid progenitor cells.

Accumulating evidence revealed the potent antitumor activities of IL-27 through multiple mechanisms, including CD8^+^ T cells, natural killer (NK) cells, antibody-dependent cell-mediated cytotoxicity, anti-angiogenesis, direct anti-proliferative effect, inhibition of expression of cyclooxygenase-2 and prostaglandin E_2_, and suppression of epithelial–mesenchymal transition, depending on the characteristics of individual tumors (43–45). One of the most critical tumor suppressors, p53, is inherently instable and mutated in approximately 50% of tumors, and various stressors such as DNA damage or oncogenic activation such as RAS mutations can affect the oncogenic properties of the mutant p53 (46). Because IL-27 possesses anti-inflammatory and antitumor properties, the role of endogenous IL-27 signaling in the mutant p53-mediated tumorigenesis was investigated (47). Lack of IL-27 signaling was shown to decrease the survival and double the incidence of osteosarcoma, possibly due to increased stability of the mutant p53 protein expression, indicating that IL-27 signaling negatively modulates the oncogenic properties of mutant p53 *in vivo* (47). In addition, lack of IL-27 signaling was demonstrated to cause spontaneous liver inflammation, fibrosis, and steatosis (48).

## Interleukin-35

**Figure 3:**
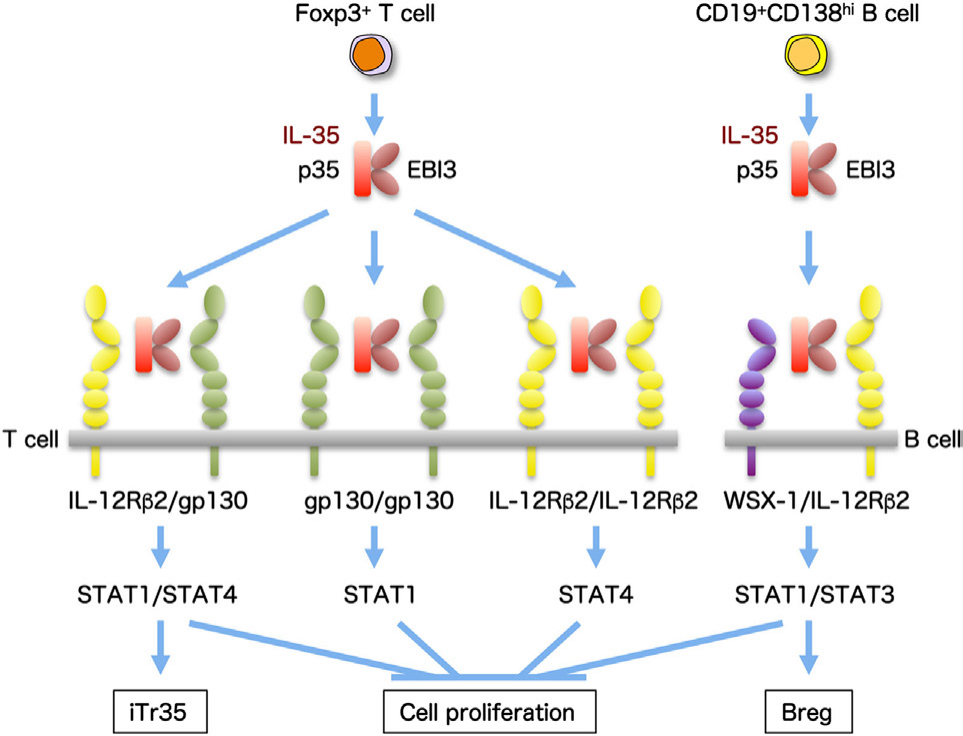
**IL-35 and the composition of its receptor subunits**. IL-35, a heterodimeric cytokine of p35 and EBI3, is preferentially secreted by forkhead box P3 (Foxp3^+^) Treg cells and induces IL-35-producing Treg (iTR35) cells with immunosuppressive activity. The receptor for IL-35 is a heterodimer of gp130 and IL-12Rβ2, and it signals *via* activation of a heterodimer of STAT1 and STAT4. In addition, IL-35 also has the ability to mediate signaling in the presence of only one of them as a homodimer. IL-35 also produced from CD19^+^CD138^hi^ B cells induces the expansion of B regulatory (Breg) cells producing IL-35 and, intriguingly, activates a heterodimer of STAT1 and STAT3 through the IL-35 receptor comprising IL-12Rβ2 and WSX-1.

Interleukin-35 is preferentially secreted by forkhead box P3^+^ Tregs and has suppressive activity (12). IL-35 also induces the conversion of conventional T cells into a suppressive IL-35-producing forkhead box P3^−^-induced Treg-cell population (termed iTr35), and this contagious spread of suppression is known as infectious tolerance (49). The receptor for IL-35 is a heterodimer of gp130 and IL-12Rβ2. In addition, IL-35 has the ability to mediate signaling in the presence of only one of them as a homodimer through activation of the heterodimer or homodimer of signal transducer and activator of transcription (STAT)1 and STAT4 (13) (Figure 3). In B cells, IL-35 was recently demonstrated to induce the expansion of a unique IL-35-producing regulatory B cell that conferred protection from experimental autoimmune uveitis (50). Intriguingly, IL-35 activates a heterodimer of STAT1 and STAT3 through the IL-35 receptor comprising the IL-12Rβ2 and WSX-1 in B cells (50). Moreover, B cell-restricted deficiency in p35 or EBI3 exacerbated experimental autoimmune encephalomyelitis and enhanced resistance to *Salmonella* infection (51).

The testis has immune privilege, and the maintenance of that immune privilege status in the testes is controlled by multiple mechanisms, including the sequestration of antigens and antibodies from the immune system by the blood–testis barrier, the immunosuppressive properties of local cells, and the production of paracrine and endocrine factors (52, 53). Expression of EBI3 was recently demonstrated to markedly increase in the testes of 10- or 12-week-old wild-type mice as compared with levels in 2-week-old mice, whereas mRNA expression of p35 was conserved between these two groups (54). Lack of EBI3, p35, and IL-12Rβ2 caused enhanced infiltration of lymphocytes into the testicular interstitium with increased IFN-γ expression in the testes and autoantibody production against mainly acrosomal regions of spermatids (54). Immunohistochemical analyses revealed that CD163^+^ resident macrophages positive for p35 and EBI3, possibly producing IL-35, were detected in the interstitium of 12-week-old mice, but not in those of 2-week-old mice (54). These results suggest that IL-35 contributes to maintaining the testicular immune privilege.

It was previously demonstrated that there are multiple IL-35^+^ cells in tumor-bearing mice and patient samples and that forced expression of IL-35 in a tumor promotes tumor progression (49, 55). Recently, the physiological impact of IL-35 on the tumor microenvironment was examined (56). Neutralization with IL-35-specific antibody or Treg cell-restricted deletion of IL-35 production limited tumor growth in multiple tumor models due to enhanced T-cell proliferation, effector function, antigen-specific responses, and long-term T-cell memory (56). Thus, IL-35 plays critical roles in preventing autoimmunity, maintaining self-tolerance, and suppressing antitumor immune responses.

## Interleukin-39

Interleukin-39 is the most recently discovered cytokine. It belongs to the IL-6/IL-12 family and consists of IL-12/IL-23p19 and IL-27/IL-35EBI3, whose stable association was demonstrated in culture supernatant of activated B cells by immunoprecipitation (14). Secretion of IL-39 by activated B cells that mediate lupus-like diseases in MRL/lpr mice was demonstrated to be significantly elevated compared to other IL-12 family cytokines. In addition, adoptive transfer of activated B cells depleted of p19 or EBI3 to mice with lupus-like disease ameliorated hallmark features of systemic lupus erythematosus, including reduction of splenomegaly, pathogenic B cells, and proteinuria (14). IL-39 was also shown to induce differentiation and/or expansion of neutrophils, whose ability is critically important for induction of pathogenic features of the autoimmune disease (57). Moreover, IL-39-induced neutrophils had positive feedback on IL-39 expression in activated B cells by secreting B-cell activation factor (57). Thus, IL-39 secreted by activated B cells may be an important pro-inflammatory cytokine and a potential therapeutic target for the treatment of autoimmune diseases such as systemic lupus erythematosus.

A similar possible association between p19 and EBI3 was suggested in damaged keratinocytes (58). In keratinocytes, toll-like receptor 3 has an important role in detecting damage-associated molecular patterns released from damaged cells and in initiating cell repair processes (59). Toll-like receptor 3-mediated activation of keratinocytes was demonstrated to drive IFN regulatory factor 6-dependent p19 expression and p19/EBI3 heterodimer formation, possibly contributing to wound healing by damping inflammatory responses (58). Further studies are necessary to clarify whether IL-39 is a pro-inflammatory cytokine or an anti-inflammatory cytokine, or both, in keratinocytes.

## IL-Y (p28/p40)

**Figure 4:**
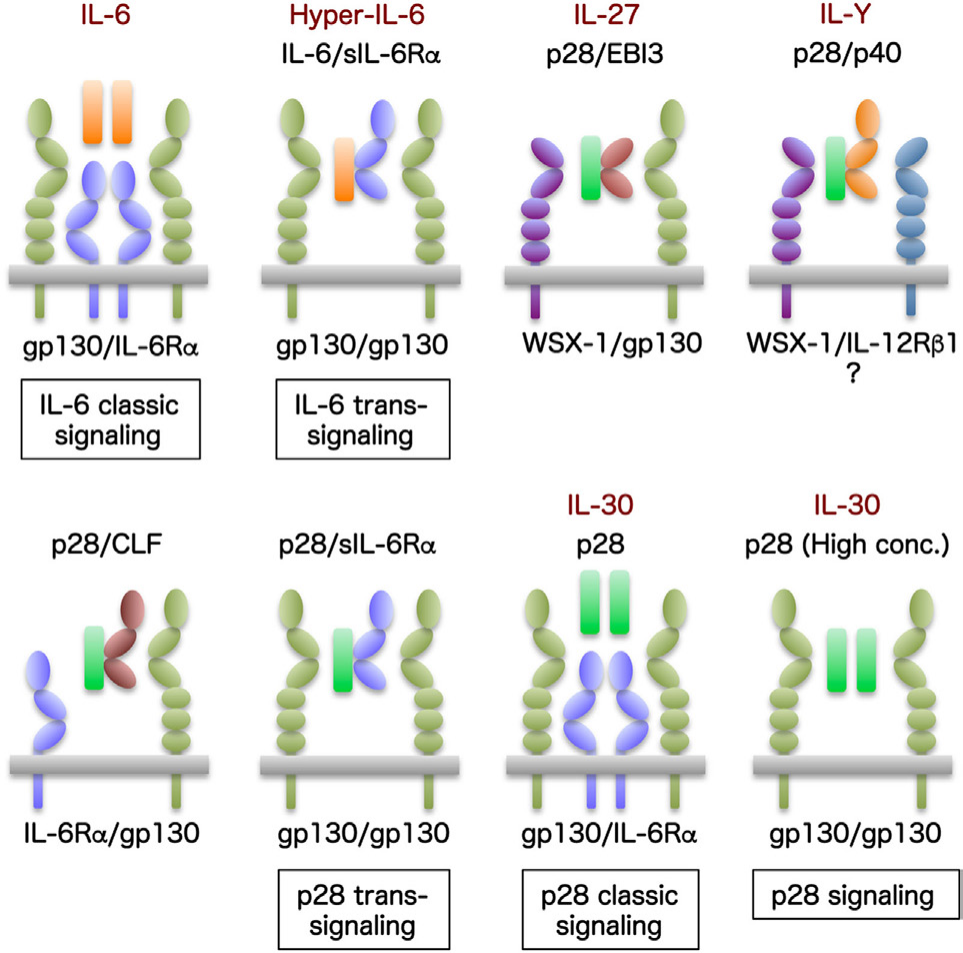
**IL-30 and its related molecules**. IL-6 is unique in signaling through not only membrane-bound IL-6Rα but also sIL-6Rα. The former is called IL-6 classic signaling through membrane-bound IL-6Rα together with gp130, and the latter is called IL-6 *trans*-signaling by IL-6 bound with sIL-6Rα and IL-6/sIL-6Rα (also designated Hyper-IL-6) through only gp130. The α subunit of IL-27, p28, which is referred to as IL-30, functions as an agonistic cytokine *via* the gp130 signaling pathway similar to IL-6, either by itself or associating with other molecules such as IL-12p40, CLF, IL-6Rα, forming p28/IL-6Rα classic signaling, p28/sIL-6Rα *trans*-signaling, and p28 signaling. Moreover, p28 can activate signal transduction *via* gp130, even in the absence of IL-6Rα or EBI3, albeit at higher concentrations.

The α-subunit of IL-27, p28, which is referred to as IL-30, was initially reported to inhibit gp130 signaling by directly binding to gp130, resulting in inhibition of Th17 differentiation as an antagonist (60). However, IL-30 was demonstrated to function as an agonistic cytokine *via* the gp130 signaling pathway similar to IL-6, either by itself or associated with other molecules, such as IL-12p40 (15, 16), cytokine-like factor 1 (CLF) (61), IL-6Rα (62), forming p28/IL-6Rα classic signaling, p28/sIL-6Rα *trans*-signaling, and p28 signaling (Figure 4).

After identification of IL-35 in 2007 (12), additional potential pairings were examined; one of these candidates was the IL-27p28/IL-12p40 heterodimer (15). Initial studies using Western blot followed by immunoprecipitation reaction revealed that a stable association between p28 and p40 was formed possibly *via* disulfide bond (15). The therapeutic effect of p28/p40 was then examined using a mouse model of experimental autoimmune uveitis, which is caused by Th1 and Th17 cells. Injection of p28/p40 protein suppressed experimental autoimmune uveitis by inhibiting the differentiation and inflammatory responses of Th1 and Th17 cells (15). These suppressive effects seemed to be ascribed to antagonizing the activation of STAT1 and STAT3 pathways induced by IL-27 and IL-6, both of which signal through the gp130 receptor (15). Moreover, recent studies using adenovirus vector expressing p28/p40 (IL-Y) suggested that treatment of prediabetic non-obese diabetic mice prevented the onset of hyperglycemia with reduced expression of inflammatory mediators such as IFN-γ (16). Interestingly, in this study, p28/p40 was demonstrated to be able to significantly stimulate a unique cytokine and chemokine expression profile as well as to activate STAT3, in part, through a pathway involving WSX-1 (16). These results implicate that the p28/p40 might be a bioactive immunosuppressive cytokine and not a mere competitive inhibitor against IL-27, IL-12, or IL-23.

## p28/CLF

Independently of EBI3, p28 also forms a complex with CLF, and the resultant p28/CLF complex is produced by activated dendritic cells (61). CLF is a soluble cytokine receptor that forms a complex with cardiotrophin-like cytokines, which have neurotrophic and immunomodulatory activities, for efficient secretion (63). p28/CLF was demonstrated to activate NK cells with IL-12-induced and IL-2-induced IFN-γ production and to induce activation of STAT1 and STAT3, which requires gp130 and IL-6Rα, but not WSX-1, as receptor subunits for signaling in CD4^+^ and CD8^+^ T cells (61). It also promoted IL-17 and IL-10 secretion from CD4^+^ T cells and inhibited their proliferation. In B cells, p28/CLF enhanced their proliferation and induced differentiation into plasma cells producing IgM, IgG2c, and IgG1 (64). In the factor-dependent B-cell line, Ba/F3 cells expressing gp130 and IL-6Rα, p28/CLF induced activation of STAT1 and STAT3 and their proliferation (61). Similar effects were also observed with p28 alone, indicating that p28 alone is biologically active in cells expressing gp130 and IL-6Rα, such as hepatocytes like IL-6 (61, 62). This is because CLF is assumed to be necessary only for secretion of p28, but not for p28/IL-6Rα-mediated signaling (61, 62).

## p28/sIL-6Rα (p28 *trans*-Signaling)

Because IL-6Rα is only expressed in hepatocytes and subtypes of lymphocytes, but gp130 is almost ubiquitously expressed, IL-6 can signal *via* not only the membrane-bound IL-6Rα and gp130 (IL-6 classic signaling) but also the sIL-6Rα through only gp130 (IL-6 *trans*-signaling); serum levels of sIL-6Rα are increased under inflammatory conditions and the spectrum of IL-6-responsive cells expands to almost all types of cells throughout the body (2, 5). Similarly, p28 can also induce signaling *via* membrane-bound IL-6Rα; therefore, p28 was speculated to induce *trans*-signaling *via* sIL-6Rα (61). Indeed, p28/sIL-6Rα fusion protein similar to IL-6/sIL-6Rα (Hyper-IL-6) was shown to induce phosphorylation of STAT3 and proliferation of Ba/F3 cells expressing gp130, which are inhibited by soluble gp130 (62). Although the findings of p28/sIL-6Rα *trans*-signaling expand the spectrum of its responsive cells to virtually all cells in the body, such as IL-6, physiological roles of the p28/sIL-6Rα *trans*-signaling remain to be clarified.

## IL-30 (p28) (p28 Classic Signaling)

Although IL-27 signals *via* a heterodimer of gp130 and WSX-1, p28/IL-6Rα was demonstrated to specifically recruit two gp130 receptors for signal transduction (62). The binding of p28 to a gp130/WSX-1 heterodimer or a gp130 homodimer was revealed to be highly selective and controlled by a novel molecular switch induced by EBI3 (as IL-27) or IL-6Rα (as p28 classic signaling), respectively (62). Moreover, because p28 has an intrinsic affinity for gp130 (60), p28 was able to activate signal transduction *via* gp130, even in the absence of IL-6Rα or EBI3, albeit at higher concentrations (62).

Recent evidence suggested that IL-30 also acts as an IL-27-independent self-standing cytokine with its own functions in addition to associating with EBI3 to form IL-27. These functions include a hepatoprotective role against liver injury and liver fibrosis induced by acute and chronic inflammation with IL-12, IFN-γ, concanavalin A, and carbon tetrachloride (65–67). This is consistent with the fact that IL-6Rα is mainly expressed on hepatocytes (4, 5, 62). Injection of the IL-30 expression vector was demonstrated to inhibit IL-12-induced and concanavalin A-induced liver injury due to suppression of IFN-γ production (65). Consistent with this, dendritic cell-specific p28 conditional knockout mice exacerbated concanavalin A-induced liver injury with higher production of IFN-γ from CD4^+^ T cells, but not NKT cells (66). In addition, injection of the IL-30 expression vector attenuated liver fibrosis by recruiting NKT cells into the liver to remove activated hepatic stellate cells through an NKG2D–Rae1 interaction (67).

Moreover, IL-30, but not EBI3, was demonstrated to be expressed in prostate cancer lesions and tumor-draining lymph nodes, such as CD68^+^ macrophages, CD33^+^/CD11b^+^ myeloid cells, and CD14 monocytes, and its expression correlated with advanced disease grade and stage (68). In addition, IL-30 stimulated proliferation of human prostate cancer cells, which express both IL-6Rα and gp130, and downregulated the expression of chemokines, such as CCL16, tumor necrosis factor superfamily member 14, and chemokine-like factor, which recruit immune cells into the tumor (68). IL-30 also upregulated the tumor suppressor and androgen co-receptor CKLF-like MARVEL transmembrane domain containing-3 and multifunctional receptor chemokine-like receptor-1 (68). Thus, IL-30 may be an important cytokine shaping the tumor and lymph node microenvironment.

## Epstein–Barr Virus-Induced Protein 3

Epstein–Barr virus-induced protein 3 was first identified in B lymphocytes as a gene whose expression is induced by Epstein–Barr virus infection and is readily secreted by itself (69). Recently, evidence revealed that EBI3 may also function as a self-standing molecule. It was demonstrated that high expression of EBI3 in lung cancer patients is associated with a poor prognosis and that serum levels of EBI3 in lung cancer patients are significantly higher than those in healthy volunteers (70). Furthermore, reduction of EBI3 expression by siRNA suppressed cancer cell proliferation and induction of exogenous EBI3 expression conferred growth-promoting activity (70).

Systemic sclerosis is a connective tissue disorder characterized by fibrosis of the skin and caused by the activation of fibroblast and excessive deposition of the extracellular matrix, mainly type I collagen (71). Expression of EBI3, but not IL-35, was recently demonstrated to be decreased in the keratinocytes of the epidermis and Treg cells of the dermis in systemic sclerosis skin compared with normal skin, whereas injection of EBI3 alone into the skin improved mice skin fibrosis and addition of EBI3 alone in cultured dermal fibroblasts decreased type I collagen expression (18). Although the possibility that EBI3 may form a complex with endogenous p35 or other molecule cannot be excluded, EBI3 itself may directly affect collagen expression.

CsEBI3, a fish EBI3 homolog, was recently identified from tongue sole (*Cynoglossus semilaevis*) and demonstrated immunostimulatory properties depending on the conserved fibronectin type III domain as a self-standing cytokine (72). Bacterial infection of peripheral blood leukocytes enhanced CsEBI3 expression and caused extracellular secretion of CsEBI3, and purified recombinant CsEBI3 stimulated the respiratory burst activity of peripheral blood leukocytes and upregulated the expression of IL-1β, IL-8, myeloid differentiation primary response gene 88, IFN-induced gene 15, CD28, and chemokines (72). Thus, the EBI3 homolog alone may play a critical role in the antimicrobial host defense in fish.

## p19

Self-standing activity of intracellular IL-23p19 was recently reported to play important roles as an endogenous activator of endothelial inflammation, promoting leukocyte adhesion to endothelial cells and transendothelial migration (19). Inflammatory mediators play important roles in the pathogenesis of vascular lesions that characterize different types of vasculitis, including giant-cell arteritis (73). It was recently demonstrated that p19 expression is enhanced by lipopolysaccharides or tumor necrosis factor-α in the absence of p40 in endothelial cells and that intracellular expression of p19 increases their cell surface expression of intracellular adhesion molecule-1 and vascular cell adhesion molecule-1, which enhance the attachment of leukocytes and increase their transendothelial migration (19). Intriguingly, the intracellular expression of p19 associated with cytokine receptor subunit gp130 and stimulated gp130-dependent activation of STAT3 signaling. In addition, endothelial p19 expression also associated with gp130 in the adventitial capillaries of inflamed temporal arteries of patients with giant-cell arteritis that do not contain p40 (19). However, how p19–gp130 interaction elicits STAT3 activation remains to be clarified.

## Conclusion and Future Perspectives

Most of the cytokines identified to date have mainly pro-inflammatory property, and there are only a few cytokines with anti-inflammatory properties, including IL-10, transforming growth factor-β, IL-27, and IL-35. Among them, IL-35 may be the only cytokine that possesses anti-inflammatory property alone. IL-10 is best known as an anti-inflammatory cytokine, but its immunosuppressive function is mainly limited to antigen-presenting cells (74). For CD8^+^ T cells and B cells, IL-10 has pro-inflammatory functions and promotes proliferation and antibody production (74). This “double-edged sword” property significantly hinders clinical application of IL-10 because such pro-inflammatory functions might lead to adverse effects. So far, only the anti-inflammatory property is known for IL-35; therefore, this cytokine could be one of the most promising candidates for clinical application against allergic and autoimmune diseases. However, among the IL-6/IL-12 family cytokines, IL-35 is considered to differ from the other cytokines (75). Although bacterially produced and purified recombinant proteins of EBI3 and p35 were correctly folded and biologically active in combination with p28 and p40, respectively, no biologically active IL-35 was reported to be formed when the p35 and EBI3 were combined (75). There are currently no reasons to explain this, but IL-35 might need additional, yet unidentified, molecules for efficient secretion and exertion of biological activity (75). One of the best criteria to prove the relationship between a bioactive cytokine and its receptor should be to examine whether the recombinant cytokine can proliferate a factor-dependent cell line such as Ba/F3 cells expressing its receptor subunits. Ba/F3 is an IL-3-dependent mouse pro-B cell line used very commonly for assessing the potency of biologically active signaling molecules, including cytokines and kinases. In addition to IL-35, IL-Y, IL-39, and new cytokines to be identified in the near future need such studies to confirm the biological relationship.

Thus, the diversity in molecular structures and functions of the IL-6/IL-12 family cytokines is still expanding in various physiological and pathological situations. Although the Human Genome Project was declared complete in 2003, and approximately 20,000 genes were identified, this number is much less than expected (76, 77). This gap is considered to be caused by the differences in subunit structure and alternative splicing. Growing diversity in the molecular structures and functions of the IL-6/IL-12 family cytokines should significantly contribute to filling the gap. Currently, the newest cytokine is IL-39, but some cytokine family members might be renamed because of new interleukins that might be found in the near future.

## Author Contributions

HH and TY organized and wrote the manuscript. IM and MX designed and drew the figures. YC and MO commented on the manuscript.

## Conflict of Interest Statement

The authors declare that the research was conducted in the absence of any commercial or financial relationships that could be construed as a potential conflict of interest. The reviewer DK and handling Editor declared their shared affiliation, and the handling editor states that the process nevertheless met the standards of a fair and objective review.

## Abbreviations

CLF, cytokine-like factor 1; EBI3, Epstein–Barr virus-induced protein 3; gp130, glycoprotein 130; IFN, interferon; Ig, immunoglobulin; IL, interleukin; NK, natural killer; R, receptor; s, soluble; STAT, signal transducer and activator of transcription; Th, helper T; Treg, regulatory T.
